# Renomegaly and acute kidney injury as primary manifestations of non-Hodgkin’s lymphoma: a report of three cases

**DOI:** 10.1186/s13000-023-01408-7

**Published:** 2023-12-08

**Authors:** Yu Bai, Yuanyuan Zheng, Qidong Zhang, Qun Jiang, Zongli Diao, Wang Guo, Sha Liu, Wenhu Liu

**Affiliations:** 1 Department of Nephrology, Faculty of Kidney Diseases, Beijing Friendship Hospital, Capital Medical University, Beijing, China; 2 Department of Pathology, Faculty of Kidney Diseases, Beijing Friendship Hospital, Capital Medical University, Beijing, China

**Keywords:** Non-Hodgkin’s lymphoma, Acute kidney injury, Bilateral kidney enlargement, Renal infiltration

## Abstract

**Background:**

In adults with non-Hodgkin's lymphoma, renal enlargement and acute kidney injury occur infrequently at first presentation, especially in T lymphocytic lymphomas.

**Case presentation:**

We report three cases of non-Hodgkin’s lymphoma with acute renal injury and bilateral renal enlargement. At diagnosis, one patient presented with an adrenal mass, one patient's lymph node biopsy was consistent with a renal biopsy, and one patient had primary renal lymphoma with no extrarenal disease. Assessment of renal pathology in Case 2 and Case 3 showed interstitial lymphocyte infiltration; the pathological types were non-Hodgkin's diffuse large B lymphoma originating from activated B cells outside germinal centers and non-Hodgkin's T-lymphoblastic lymphoma/leukemia, respectively. Case 1 did not receive anti-lymphoma therapy and died from infection and multiple organ failure within 1 month of hospitalization. Case 2 received eight courses of R-CHOP; her lymphoma recurred 2 years after diagnosis and she died from severe pulmonary infection 3 years after diagnosis. Case 3 received hyper-CVAD regularly and achieved stable renal function; this patient remains under follow-up.

**Conclusions:**

Renal lymphoma may have diverse manifestations, especially primary renal lymphoma without extrarenal involvement. Nephrologists should pay careful attention to these manifestations to ensure accurate diagnosis.

## Background

Renal involvement in non-Hodgkin's lymphoma (NHL) is relatively common. Previous studies have suggested that up to 10% of patients with NHL and lymphocytic leukemia may develop kidney injury [1]. Lymphoma is associated with various forms of renal involvement, including primary renal lymphoma, other lymphomas involving the kidney, and secondary renal injury caused by therapeutic drugs and tumor lysis. The complexity of distinguishing primary and secondary renal involvement increases the difficulty of diagnosis [2]. Therefore, in clinical practice, diagnosis of renal involvement in lymphoma is challenging for nephrologists.

Acute renal injury (AKI) is common in patients with NHL and lymphocytic leukemia, but it is less common in patients with acute renal injury who initially present in nephrology departments [3]. Here, we report three cases of NHL with renomegaly and acute renal injury as the primary manifestations of disease.

## Report of cases

### Case 1

A 57-year-old woman presented with abdominal pain for 20 days and anuria for 3 days. Increasing creatinine level within 1 month indicated AKI and the patient was treated by urgent dialysis in the emergency department. The changes of serum creatinine and urine volume are shown in Table 1. The patient had a 10-year history of type 2 diabetes (treated with acarbose) as well as a 2-year history of depression (treated with paroxetine and olanzapine). The patient had no history of hypertension. Her blood pressure (BP) was 140/80 mmHg and her heart rate (HR) was 110 beats per minute (bpm). Cardiopulmonary and abdominal examinations showed normal results. The results of laboratory examinations are shown in Table 2. Abdominal ultrasound showed intrahepatic patchy hypoechoic area and splenomegaly. Both kidneys were enlarged with echo enhancement: the left kidney was 15.8 × 7.0 × 8.2 cm in size and the right kidney was 16.3 × 7.8 × 8.5 cm in size. Multiple hypoechoic areas were observed in both kidneys. A 6.3 × 2.6 cm hypoechoic nodule in the right adrenal region had clear boundaries and dotted blood flow signals indicating the presence of a tumor. An abdominal computed tomography (CT) scan showed that the right adrenal mass and the right liver lobe was invaded. In addition, multiple nodules in both kidneys with retroperitoneal lymphadenopathy and splenomegaly were considered to represent lymphoma with multiple metastases. Ultrasound-guided puncture biopsy of the right adrenal mass was performed. Large numbers of lymphocytes had infiltrated into the tissue of the right adrenal gland tumor the infiltrating cells were medium or large, round, had oval nuclei, and were slightly irregular in shape. Immunohistochemistry showed that the neoplastic lymphoid cells were positive for leukocyte common antigen (LCA), B-cell lymphoma 2 (Bcl-2), CD21, CD20 (diffuse), Bcl-6, melanoma ubiquitous mutated protein 1 (Mum-1), melanoma antigen recognized by T cells 1 (Mart-1), and Ki-67 (> 70%) but negative for inhibin, S-100, CD5, CD10, transcription initiation factor 1 (TIF-1), CD3, glypican 3 (GPC-3), and chromogranin A (CgA). The patient was diagnosed with non-germinal center B-cell-like diffuse large B lymphoma and renal biopsy was not performed because of the poor general condition of the patient and increasing bleeding risk. After renal replacement therapy and supportive therapy, the patient’s renal function gradually recovered (urine volume increased to 1500 mL/day and creatinine level gradually decrease to 180 µmol/L). Unfortunately, because of systemic lymphoma metastasis and poor general condition, the patient was unable to receive anti-tumor treatment and died from infection and multiple organ failure within 1 month of hospitalization.

**Table1:** Diagnosis of AKI in three cases of non-Hodgkin’s lymphoma

	case1	case2	case3
Initial Scr (μmol/l)	65	218	292.3
Scr near admission (μmol/l)	966	329.6	541.2
Interval (days)	17	5	2
Urine volume	Anuria	Oliguria	Normal
Duration (days)	3	1	-

*Scr* Serum creatinine. Interval refers to the interval of the change of serum creatinine. Duration refers to the duration of urine volume status

**Table 2:** Results of laboratory analyses for three cases of non-Hodgkin’s lymphoma

	case1	case2	case3
Age(year)/sex	57/F	48/F	32/M
Serum albumin (g/L)	25	30	46.1
Serum creatinine (μmol/L)	1097	419	541.2
Urea nitrogen (mmol/L)	22.52	11.23	22.99
Uric Acid (μmol/L)	666	548	1962.6
Serum potassium (mmol/L)	6.85	4.18	6.12
Serum phosphorus (mmol/L)	1.7	1.47	2.38
LDH (U/L)	701	504	238
PTH (pg/ml)	NA	121.7	265.2
WBC (*10^9^/L)	8.69	4.5	6.42
Hemoglobin (g/L)	131	81	113
Platelet (*109/L)	173	132	156
Urine protein	1 +	2 +	-

*Abbreviations*: *PTH* Parathyroid hormone, *LDH* Lactic dehydrogenase, *WBC* White blood cell

### Case 2

**Fig. 1:**
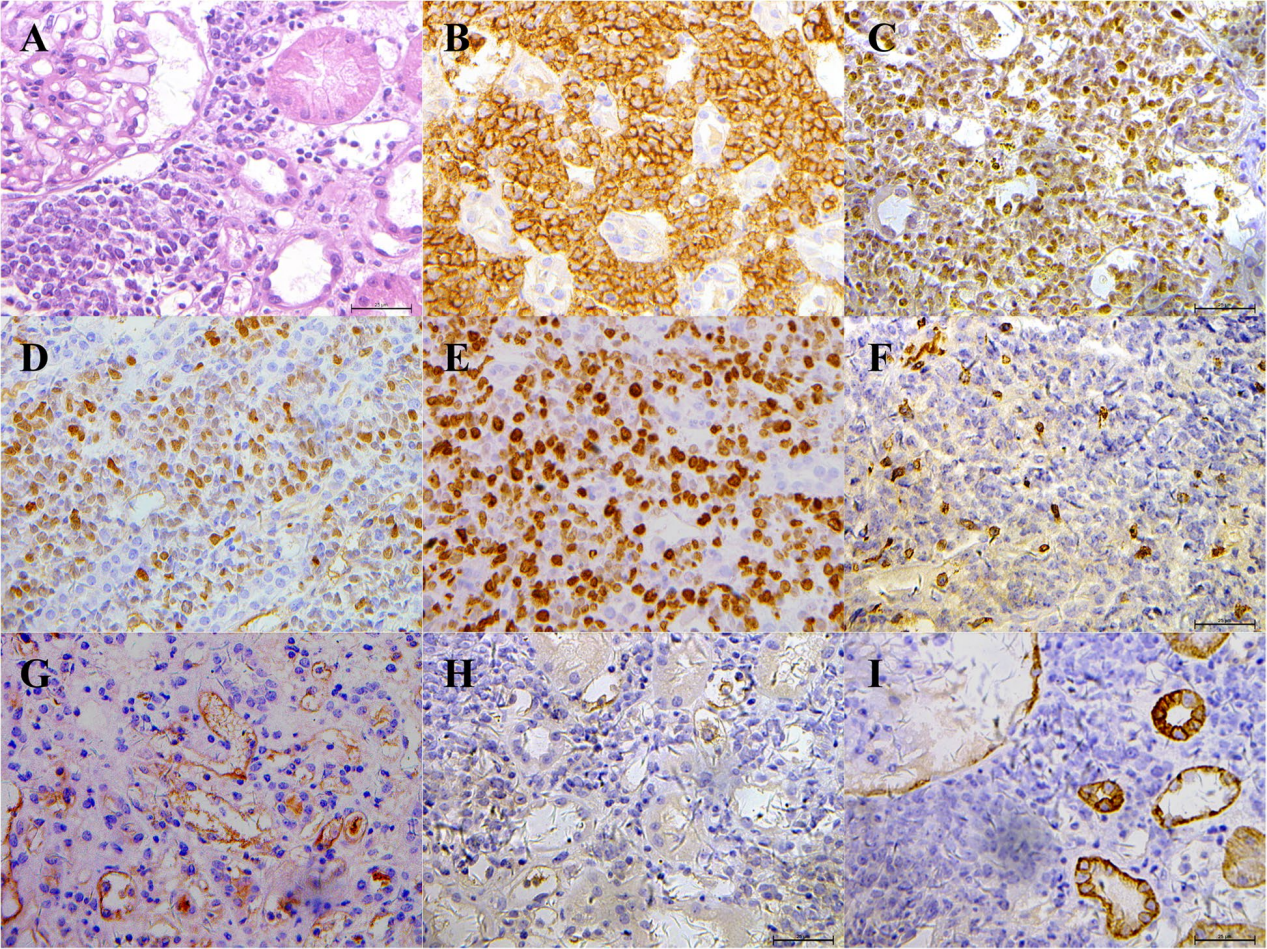
Kidney pathology of Case 2. **A** Kidney pathology showed a massive interstitial infiltration of lymphocytes (hematoxylin and eosin). **B-E** Immunohistochemical stains were positive for (**B**) CD20, (**C**) BCL-6, (**D**) Mum-1, (**E**) Ki-67 (> 50%), and negative for (**F**) CD3, (**G**) CD10, (**H**) CD21, (**I**) CK. (Original maginification × 200)

A 48-year-old woman presented with abdominal pain and AKI (Table 1). The patient had a history of appendicectomy (30 years previously) and two caesarean Sects. (17 and 25 years ago). She had no history of hypertension, diabetes, or other chronic diseases. Her BP was 170/95 mmHg and her HR was 110 bpm. Physical examination showed a pale appearance, tenderness in the right upper quadrant, and percussion tenderness over the kidney region. Cardiopulmonary examinations were normal. The results of laboratory examinations are shown in Table 2. Renal ultrasound showed bilateral renal enlargement with echo enhancement; the right kidney was 15.3 × 6.4 cm in size and the left kidney was 15.1 × 6.8 cm in size. An abdominal CT scan revealed significantly bilateral enlargement of the kidneys, multiple perinephric lymph nodes, and small retroperitoneal lymph nodes. Bone marrow biopsy findings were unremarkable. Kidney pathology showed massive and monomorphic interstitial infiltration of lymphocytes that were morphologically indistinguishable (Fig. 1A).

**Fig. 2:**
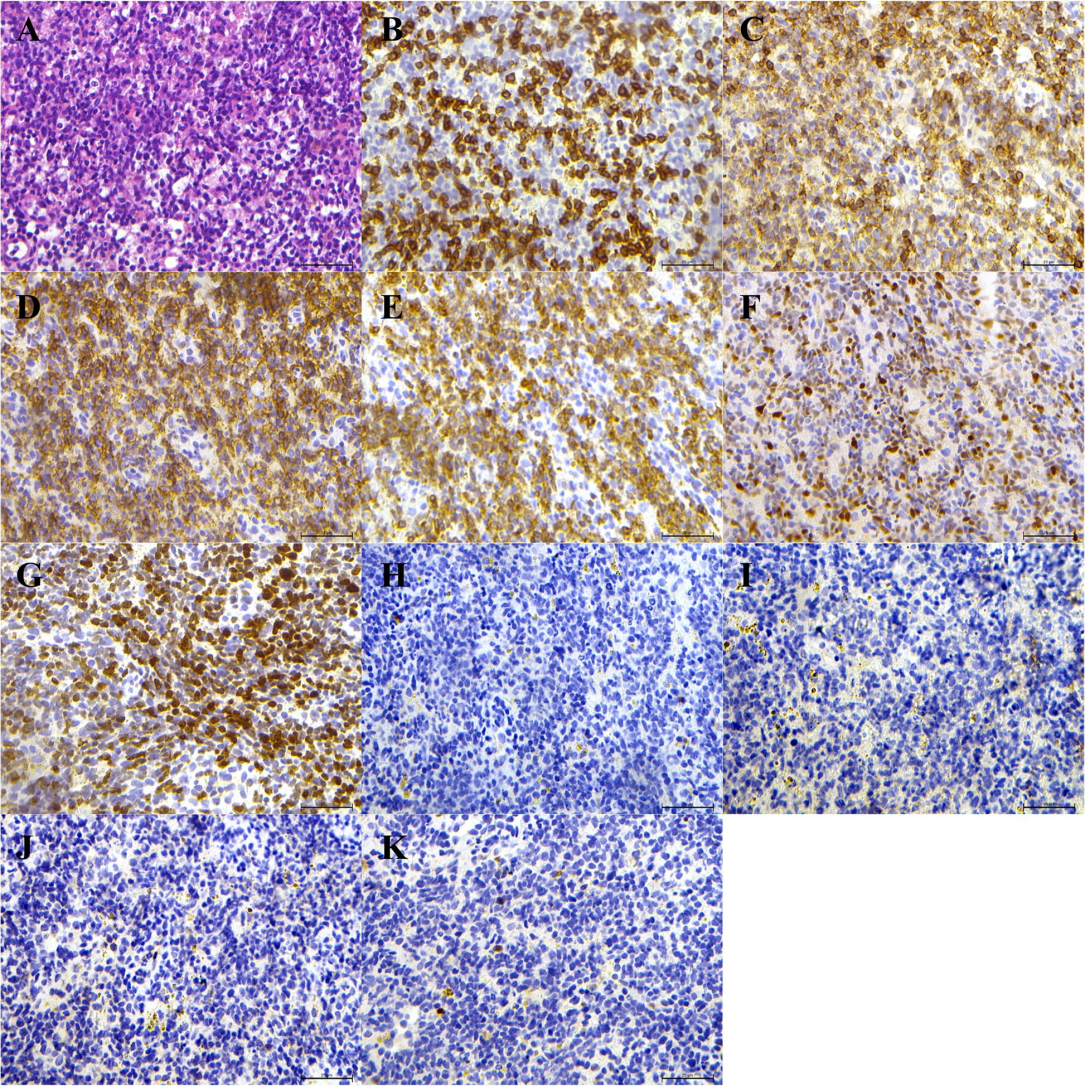
Left axillary lymph node pathology of Case 2. **A** There was diffuse proliferation of atypical lymphocytes in the lymph nodes, and the cells were of medium size with irregular nuclei (hematoxylin and eosin). **B-G** Immunohistochemical stains were positive for (**B**) CD3 (positive for a small number of cells), (**C**) CD5 (weakly positive), (**D**) CD20, (E) BCL-2, (**F**) MUM-1, (**G**) Ki-67 (> 50%), and negative for (**H**) CD10, (**I**) CD21, (**J**) CD23, (**K**) Cyclin D1.(Original maginification × 200)

Immunohistochemical staining was positive for CD20, Bcl-6, Mum-1, and Ki-67 (> 50%) (Fig. 1B–E) but negative for CD3, CD10, CD21, and creatinine kinase (CK) (Fig. 1F–I). The patient was diagnosed with non-Hodgkin's diffuse large B lymphoma originating from activated B cells outside of germinal centers. Left axillary lymph node pathology showed diffuse proliferation of atypical lymphocytes in the lymph nodes; the infiltrating cells were of medium size with irregular nuclei (Fig. 2A).

Immunohistochemical staining was positive for CD3 (staining of a small number of cells), CD5 (weakly positive), CD20, Bcl-2, Mum-1, and Ki-67 (> 50%) (Fig. 2B–G), and negative for CD10, CD21, CD23, and Cyclin D1 (Fig. 2H–K), consistent with the kidney pathology findings. After admission, the patient received supportive therapy including antihypertensive agents, sodium polystyrene sulfonate, febuxostat, and diuretics. Following diagnosis with non-Hodgkin's diffuse large B lymphoma originating from activated B cells outside of germinal centers the patient received eight courses of rituximab, cyclophosphamide, hydroxydaunorubicin, oncovin, and prednisone (R-CHOP). Her serum creatine decreased to 156 μmol/L after anti-lymphoma therapy. Her lymphoma recurred 2 years after diagnosis and she was sequentially treated with cyclophosphamide, vincristine, and prednisone (COP) and dexamethasone, ifosfamide, cisplatin, and etoposide (DICE) for 1 year. The patient died from severe pulmonary infection 3 years after diagnosis.

### Case 3

**Fig. 3:**
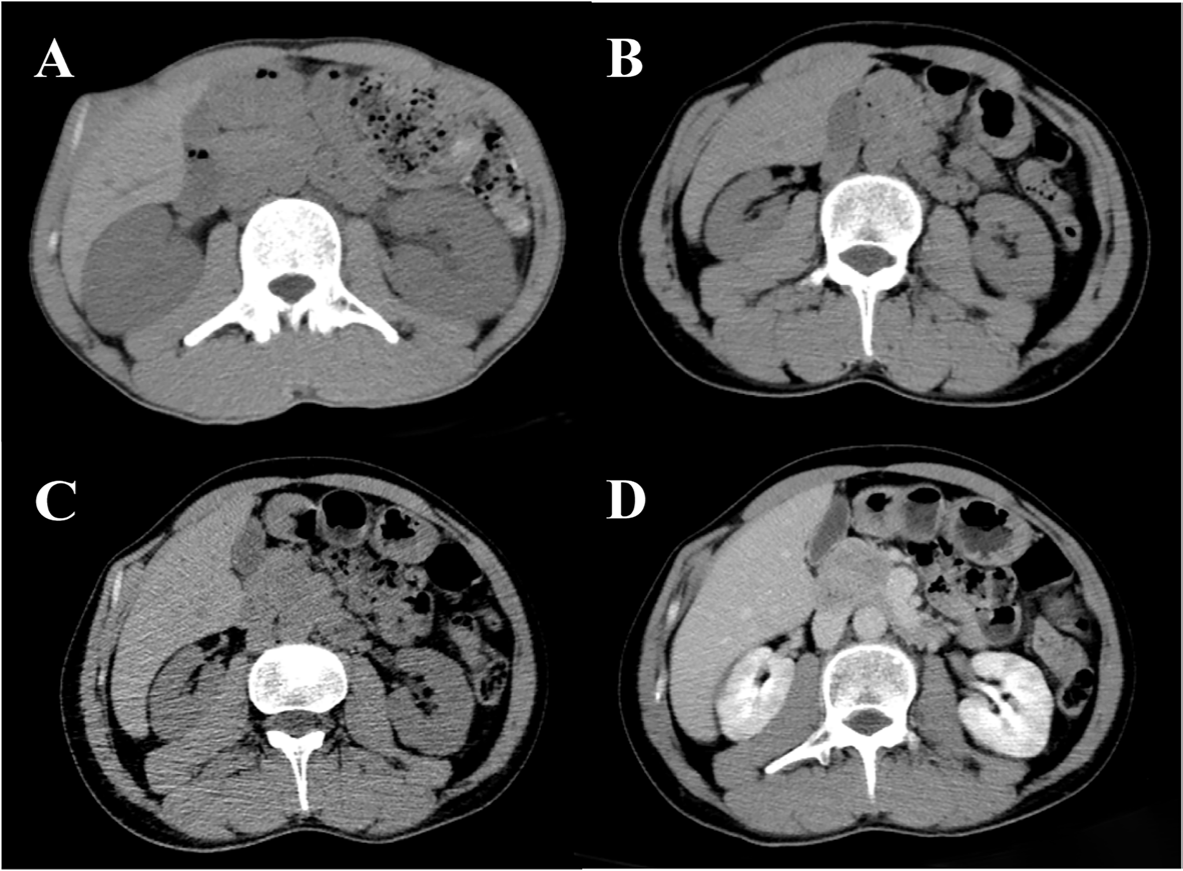
Abdominal computed tomography of Case 3. **A** August 2020, **B** November 2020, **C** Jaunary 2021, **D** Jaunary 2021 (enhanced)

A 32-year-old man presented as an outpatient with AKI (Table 1). His urine was cloudy without changes in urine volume and urinalysis was normal. The patient had no prior medical history. On physical examination, slight enlargement of cervical lymph nodes and bilateral ureter tenderness were observed. Cardiopulmonary examinations were normal. The results of laboratory examinations are shown in Table 2. An abdominal CT scan revealed symmetrical bilateral enlargement of the kidneys (Fig. 3).

**Fig. 4:**
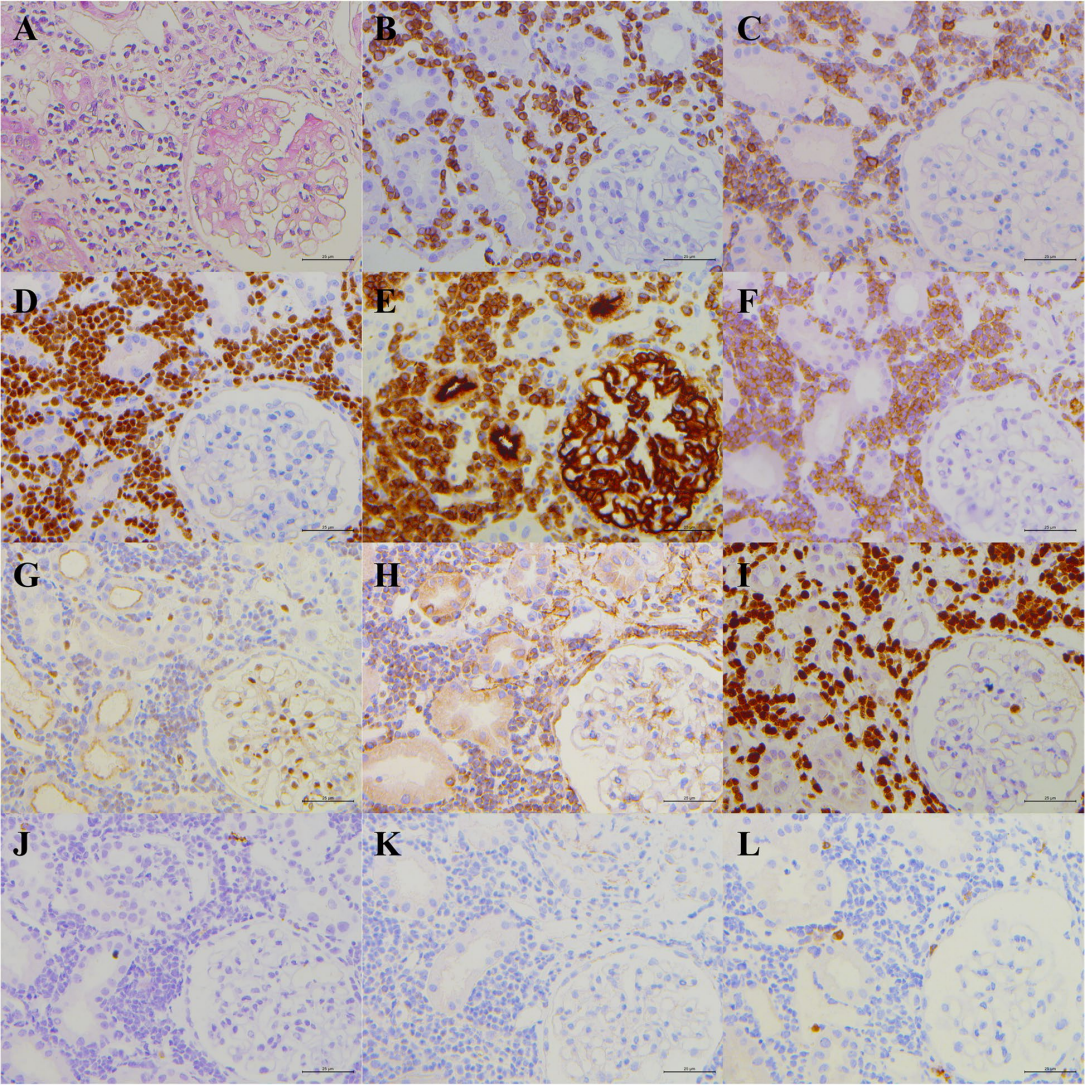
Kidney pathology of Case 3. **A** Kidney pathology shows a massive interstitial infiltration (hematoxylin and eosin). **B-I** Immunohistochemical stains were positive for (**B**) CD3, (**C**) CD5, (**D**) TDT, (**E**) CD10, (**F**) CD38, (**G**) LMO2 (weakly positive), (**H**) BCL-2 (weakly positive), (**I**) Ki-67 (80%) and negative for (**J**) CD20, (**K**) PAX-5, (**L**) MPO. (Original manification × 200)

Kidney ultrasound showed bilateral enlargement of the kidneys (right kidney 13.6 × 5.0 cm, left kidney 14.0 × 5.6 cm) with enhanced echo and bilateral renal calculi. The patient received sodium polystyrene sulfonate, febuxostat, and fluid infusions. His serum creatine and urea levels normalized after supportive therapy. The results of bone marrow biopsy and cervical lymph node biopsy were normal. Although the patient was responding well to treatment, renal biopsy was performed because of unexplained hyperuricemia. The patient was diagnosed with non-Hodgkin's T-lymphoblastic lymphoma/leukemia based on renal pathology. Analysis of kidney pathology showed massive interstitial infiltration of lymphocytes, which were similar in morphology to those of Case 2. Immunohistochemical staining was positive for CD3, CD5, terminal deoxynucleotide transferase (TdT), CD10, CD38, LIM domain only protein 2 (LMO2) (weakly positive), Bcl-2 (weakly positive), and Ki-67 (80%) but negative for CD20, paired box protein 5 (PAX-5), and myeloperoxidase (MPO) (Fig. 4).

The patient received hyper-CVAD (cyclophosphamide, vincristine, adriamycin, and dexamethasone) regularly and maintained stable renal function. A recent abdominal CT scan revealed multiple solid nodules in both kidneys, which we considered to represent lymphoma (Fig. 3).

## Discussion and conclusions

The causes of lymphoma-associated AKI are diverse, including direct tumor invasion, obstructive nephropathy, ischemic nephropathy caused by tumor compression, immune mediated renal injury [4], renal injury caused by tumor metabolism (tumor lysis syndrome), and treatment-related renal injury [5]. We reported a total of 3 lymphoma patients who presented to the nephrology department with AKI and bilateral renal enlargement as the first manifestations of disease. Findings were suggestive of lymphomatous infiltration of the kidneys [6]. The incidence of renal infiltration in NHL is more than 50% [1], including single (10%–20%) or multifocal nodules (60%), renal invasion from contiguous retroperitoneal disease (25%–30%), diffuse infiltration (20%), or perirenal involvement (10%) [7, 8].  And the primary NHL of the kidney is very rare, accounting for only 0.7% to 1.0% of lymphoma with renal involvement [9], as in case 3.

The mechanism of AKI in Case 1 was obscure because of the patient’s poor general condition and the absence of pathological evidence in the kidney. Both Case 2 and Case 3 underwent renal biopsy, which showed diffuse interstitial infiltration of the kidney by lymphoma cells. However, the mechanisms of AKI in these two cases were quite different. In Case 2, we inferred that the cause of AKI was direct damage to the kidney caused by diffuse lymphoma cell infiltration because levels of uric acid were not significantly increased, there were no signs of urinary tract obstruction, and renal function did not recover after supportive treatment and only gradually recovered after chemotherapy. In Case 3, AKI may have resulted from tumor lysis syndrome (TLS) and direct injury associated with primary renal lymphoma. However, the renal function of Case 3 returned to normal prior to chemotherapy following symptomatic treatment to lower uric acid levels and achieve rehydration. This suggested that AKI was caused by spontaneous TLS in this patient. The incidence of spontaneous TLS in patients with T-lymphoblastic lymphoma is higher than that of other lymphomas [10–12]. According to the previous reports, the clinical features of TLS were hyperuricemia, hyperphosphatemia, and hyperkalemia, leading to AKI [13]. In addition, the elevation of lactic dehydrogenase (LDH) is also an important feature of TLS [14]. However, the LDH level in case 3 is not significantly elevated. Except for increasing LDH level, other manifestations in case 3 are consistent with TLS [15]. We speculate that the increase of LDH in this patient occurred in the early stage of tumor lysis, which is not detected due to obscure onset. And the LDH level of the patient might have fallen back at the time of admission. Unfortunately, it is difficult to trace this suspicion.

For renal metastatic tumors, enhanced CT is the preferred diagnostic method [16]. The three cases reported here had common renal radiographic features including bilateral renal enlargement. However, enhanced CT should be used with caution in the diagnosis of renal lymphoma because it may not sensitively detect loss of renal function and kidney-infiltrating lesions, and may result in additional kidney damage by contrast agents. Magnetic resonance imaging can prevent contrast agent kidney damage, but may still not be able to distinguish renal infiltration of lymphoma from collecting duct or medullary carcinoma of the kidneys, transitional cell carcinoma, or severe pyelonephritis [17]. In renal infiltration of lymphoma (Case 2 and Case 3), imaging is often not sensitive. Especially for patients with negative lymph node and bone marrow biopsies, renal biopsy offers enhanced sensitivity for diagnosis of lymphoma in its early stages [18, 19]. Therefore, we believe that for patients with AKI, bilateral symmetric renal enlargement, or unexplained hyperuricemia, renal biopsy should be performed as soon as possible even if renal function has already recovered. In addition, when infiltration of the renal interstitium by homogeneous lymphocytes is observed, nephrologists should consider the possibility of lymphoma.

The treatment and prognosis of NHL are closely related to the underlying pathological type. Non-Hodgkin's diffuse large B lymphoma accounts for about one-third of all NHLs and is the most common histologic subtype. R-CHOP is the standard treatment for patients with diffuse large cell lymphoma. The prognosis of patients with diffuse large cell lymphoma is usually assessed using the International Prognostic Index (IPI) [20]. According to the IPI, age of more than 60 years, elevated serum lactate dehydrogenase levels, advanced disease, and poor performance status are negative prognostic factors [21]. Case 2 was diagnosed with non-Hodgkin’s diffuse large B lymphoma originating from activated B cells outside the germinal center and received eight courses of R-CHOP, followed by COP and DICE after recurrence. The IPI score of Case 2 suggested a poor prognosis. T-cell lymphoblastic lymphoma is an aggressive malignancy caused by precursor T cells that occurs primarily in adolescents and young adults and accounts for approximately 2% of all patients with NHL [22]. To our knowledge, this report describes the first case of adult renal T-cell lymphoblastic lymphoma with AKI and hyperuricemia as the primary manifestations without extrarenal involvement. Case 3 was diagnosed with non-Hodgkin's T-lymphoblastic lymphoma/leukemia and received hyper-CVAD, which is widely used in the treatment of lymphoblastic lymphoma [23]. According to previous reports [24, 25], the prognosis of lymphoma with renal infiltration is poor. Commonly used prognostic factors, such clinical examination, imaging findings, and specific molecular biomarkers, have not shown consistent performance. Prognostic factors need to be further explored [26].

In conclusion, the three cases described here highlighted the lymphoma cells infiltration in kidney is a common variant of renal involvement in lymphomas, which should be considered in patients presenting with AKI and nephromegaly, and kidney biopsy should be performed to enable early diagnosis and treatment. Renal lymphoma is common, but its manifestations and mechanisms can vary significantly. Based on the three cases described here, clinicians should consider the possibility of interstitial infiltration of renal lymphoma. The complexity of diagnosis poses a challenge to nephrologists, and accurate diagnosis requires multidisciplinary collaborations.

## Data Availability

All data generated or analysed during this study are included in this published article.

## Abbreviations

NHL: Non-Hodgkin's lymphoma
AKI: Acute renal injury
PTH: Parathyroid hormone
LDH: Lactic dehydrogenase
WBC: White blood cell
CT: Computed tomography
TLS: Tumor lysis syndrome
IPI: International prognostic index
T-LBL: T-cell lymphoblastic lymphoma
